# 2,4-Dibromo­naphthalen-1-ol

**DOI:** 10.1107/S160053681103011X

**Published:** 2011-07-30

**Authors:** Abdul Rauf Raza, Aeysha Sultan, M. Nawaz Tahir

**Affiliations:** a University of Sargodha, Department of Chemistry, Sargodha, Pakistan; b University of Sargodha, Department of Physics, Sargodha, Pakistan

## Abstract

In the essentially planar (r.m.s. deviation = 0.023 Å) title compound, C_10_H_6_Br_2_O, an intra­molecular O—H⋯Br hydrogen bond generates an *S*(5) ring. In the crystal, mol­ecules are linked by an ⋯O—H⋯O—H⋯O— *C*(2) chain extending along [100], which involves the same H atom that participates in the intra­molecular hydrogen bond. Aromatic π–π inter­actions [centroid–centroid separation = 3.737 (4) Å] help to consolidate the packing.

## Related literature

For a related structure, see: Chanh *et al.* (1973 ▶): For graph-set notation, see: Bernstein *et al.* (1995 ▶).
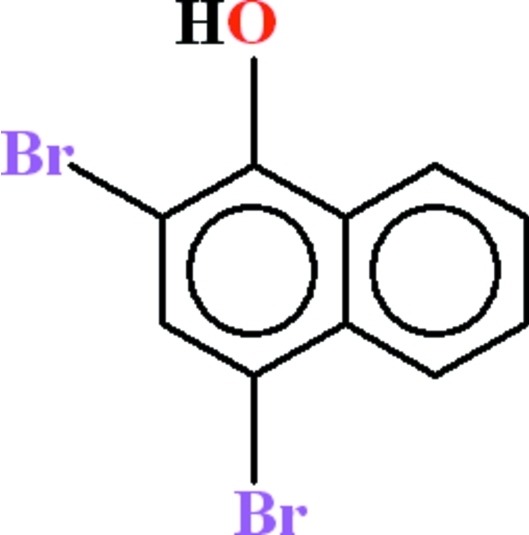


## Experimental

### 

#### Crystal data

C_10_H_6_Br_2_O
*M* *_r_* = 301.97
Orthorhombic, 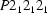

*a* = 4.1225 (3) Å
*b* = 14.4441 (11) Å
*c* = 16.0490 (14) Å
*V* = 955.65 (13) Å^3^
*Z* = 4
Mo *K*α radiation
μ = 8.44 mm^−1^
*T* = 296 K
0.32 × 0.14 × 0.12 mm

#### Data collection

Bruker Kappa APEXII CCD diffractometer
Absorption correction: multi-scan (*SADABS*; Bruker, 2005 ▶) *T*_min_ = 0.254, *T*_max_ = 0.365
5060 measured reflections
2239 independent reflections
1410 reflections with *I* > 2σ(*I*)
*R*_int_ = 0.045

#### Refinement

*R*[*F* ^2^ > 2σ(*F* ^2^)] = 0.042
*wR*(*F* ^2^) = 0.089
*S* = 0.96
2239 reflections
119 parameters
H-atom parameters constrained
Δρ_max_ = 0.47 e Å^−3^
Δρ_min_ = −0.41 e Å^−3^
Absolute structure: Flack (1983 ▶), 863 Friedel pairs
Flack parameter: −0.01 (3)

### 

Data collection: *APEX2* (Bruker, 2009 ▶); cell refinement: *SAINT* (Bruker, 2009 ▶); data reduction: *SAINT*; program(s) used to solve structure: *SHELXS97* (Sheldrick, 2008 ▶); program(s) used to refine structure: *SHELXL97* (Sheldrick, 2008 ▶); molecular graphics: *ORTEP-3* (Farrugia, 1997 ▶) and *PLATON* (Spek, 2009 ▶); software used to prepare material for publication: *WinGX* (Farrugia, 1999 ▶) and *PLATON*.

## Supplementary Material

Crystal structure: contains datablock(s) global, I. DOI: 10.1107/S160053681103011X/hb6335sup1.cif

Structure factors: contains datablock(s) I. DOI: 10.1107/S160053681103011X/hb6335Isup2.hkl

Supplementary material file. DOI: 10.1107/S160053681103011X/hb6335Isup3.cml

Additional supplementary materials:  crystallographic information; 3D view; checkCIF report

## Figures and Tables

**Table 1:** Hydrogen-bond geometry (Å, °)

*D*—H⋯*A*	*D*—H	H⋯*A*	*D*⋯*A*	*D*—H⋯*A*
O1—H1⋯Br1	0.82	2.60	3.107 (5)	122
O1—H1⋯O1^i^	0.82	2.21	2.893 (6)	141

Symmetry code: (i) 
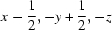
.

**Fig. 1.**
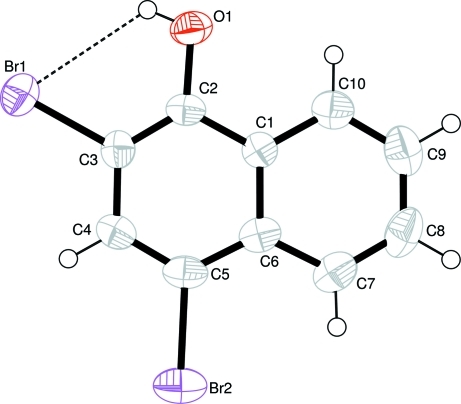
View of the title compound with displacement ellipsoids drawn at the 50% probability level. The dotted line indicates the intramolecular H-bond.

**Fig. 2.**
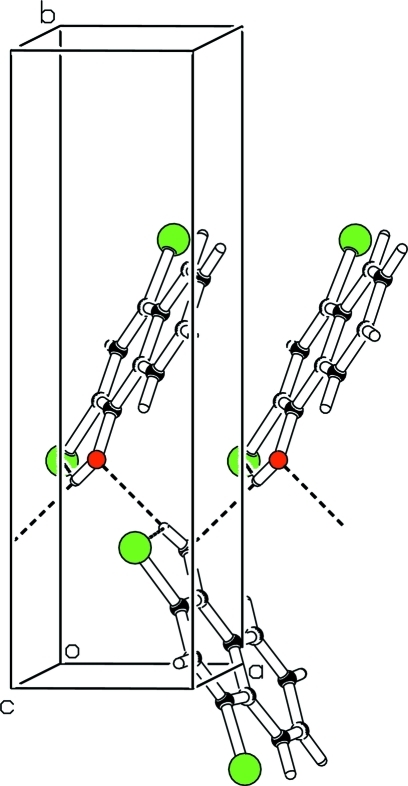
The partial packing of (I), which shows that molecules form polymeric chains.

## supplementary crystallographic information

### Comment

The crystal structure of 2-bromonaphthalene (Chanh, *et al.*, 1973) has been published which is related to the title compound (Fig. 1).

The molecule of the title compound is planar with r.m.s. deviation of 0.0234 Å. The Br2 atom has maximum deviation from the mean plane and its value is 0.0574 (27) Å. There exists an intra-molecular hydrogen bond of O—H···Br type (Table 1, Fig. 1) and complete S(5) ring motif (Bernstein *et al.*, 1995). The molecules are stabilized in the form of polymeric chains due to intermolecular H-bonding of O—H···O type (Table 1, Fig. 2). Due to these hydrogen bonds a chain of ···O—H···O—H···O— exists. The π–π interactions between the benzene rings (C1—C6) and (C1/C6—C10) of the naphthalen group at a distance of 3.737 (4) Å help to consolidate the packing.

### Experimental

Bromine (2.9 ml, 9.2 g, 30 mmol, 2 eq) was added as drops to an ice-chilled solution of α,β-unsaturated-1-tetralone (2.2 g, 15 mmol, 1 eq) in CHCl_3_ (50 ml) and was stirred for 1 h. Et_3_N (3 ml, 2.2 g, 22 mmol, 1.5 eq) was added to the reaction mixture followed by 2 h stirring at room temperature. After the commencement of reaction, the reaction mixture was neutralized with aq HCl (15 ml). The organic layer was washed with H_2_O (3 × 25 ml), dried over anhydrous Na_2_SO_4_ and concentrated under reduced pressure to afford the colorless needles of (I).

Yield: 2.4 g, 52%, m.p. 499 K.

### Refinement

The H-atoms were positioned geometrically with (O–H = 0.82, C–H = 0.93 Å) and refined as riding with *U*_iso_(H) = *xU*_eq_(C, O), where *x* = 1.5 for hydroxy and *x* = 1.2 for aryl H-atoms.

### Crystal data

**Table:**

C_10_H_6_Br_2_O	*F*(000) = 576
*M**_r_* = 301.97	*D*_x_ = 2.099 Mg m^−^^3^
Orthorhombic, *P*2_1_2_1_2_1_	Mo *K*α radiation, λ = 0.71073 Å
Hall symbol: P 2ac 2ab	Cell parameters from 1410 reflections
*a* = 4.1225 (3) Å	θ = 2.8–27.9°
*b* = 14.4441 (11) Å	µ = 8.44 mm^−^^1^
*c* = 16.0490 (14) Å	*T* = 296 K
*V* = 955.65 (13) Å^3^	Needle, colorless
*Z* = 4	0.32 × 0.14 × 0.12 mm

### Data collection

**Table:**

Bruker Kappa APEXII CCD diffractometer	2239 independent reflections
Radiation source: fine-focus sealed tube	1410 reflections with *I* > 2σ(*I*)
graphite	*R*_int_ = 0.045
Detector resolution: 7.60 pixels mm^-1^	θ_max_ = 27.9°, θ_min_ = 2.8°
ω scans	*h* = −5→5
Absorption correction: multi-scan (*SADABS*; Bruker, 2005)	*k* = −15→19
*T*_min_ = 0.254, *T*_max_ = 0.365	*l* = −20→20
5060 measured reflections	

### Refinement

**Table:**

Refinement on *F*^2^	Secondary atom site location: difference Fourier map
Least-squares matrix: full	Hydrogen site location: inferred from neighbouring sites
*R*[*F*^2^ > 2σ(*F*^2^)] = 0.042	H-atom parameters constrained
*wR*(*F*^2^) = 0.089	*w* = 1/[σ^2^(*F*_o_^2^) + (0.0309*P*)^2^] where *P* = (*F*_o_^2^ + 2*F*_c_^2^)/3
*S* = 0.96	(Δ/σ)_max_ < 0.001
2239 reflections	Δρ_max_ = 0.47 e Å^−^^3^
119 parameters	Δρ_min_ = −0.41 e Å^−^^3^
0 restraints	Absolute structure: Flack (1983), 863 Friedel pairs
Primary atom site location: structure-invariant direct methods	Flack parameter: −0.01 (3)

### Special details

**Table:**

Geometry. Bond distances, angles *etc*. have been calculated using the rounded fractional coordinates. All su's are estimated from the variances of the (full) variance-covariance matrix. The cell e.s.d.'s are taken into account in the estimation of distances, angles and torsion angles
Refinement. Refinement of *F*^2^ against ALL reflections. The weighted *R*-factor *wR* and goodness of fit *S* are based on *F*^2^, conventional *R*-factors *R* are based on *F*, with *F* set to zero for negative *F*^2^. The threshold expression of *F*^2^ > σ(*F*^2^) is used only for calculating *R*-factors(gt) *etc*. and is not relevant to the choice of reflections for refinement. *R*-factors based on *F*^2^ are statistically about twice as large as those based on *F*, and *R*- factors based on ALL data will be even larger.

### Fractional atomic coordinates and isotropic or equivalent isotropic displacement parameters (Å^2^)

**Table:**

	*x*	*y*	*z*	*U*_iso_*/*U*_eq_	
Br1	0.45967 (15)	0.18900 (4)	0.18041 (4)	0.0493 (2)	
Br2	1.06884 (18)	−0.15734 (4)	0.19773 (5)	0.0624 (3)	
O1	0.6998 (11)	0.1798 (3)	−0.0029 (3)	0.0463 (16)	
C1	0.9478 (14)	0.0326 (3)	0.0024 (3)	0.0333 (17)	
C2	0.7730 (14)	0.1034 (4)	0.0449 (4)	0.0343 (19)	
C3	0.6882 (14)	0.0934 (4)	0.1244 (4)	0.036 (2)	
C4	0.7718 (13)	0.0149 (4)	0.1698 (4)	0.039 (2)	
C5	0.9399 (15)	−0.0536 (4)	0.1318 (4)	0.0417 (19)	
C6	1.0379 (14)	−0.0490 (4)	0.0474 (3)	0.0377 (19)	
C7	1.2132 (16)	−0.1172 (4)	0.0024 (5)	0.048 (3)	
C8	1.2958 (16)	−0.1080 (5)	−0.0777 (4)	0.058 (3)	
C9	1.2149 (16)	−0.0287 (5)	−0.1202 (5)	0.059 (3)	
C10	1.0428 (15)	0.0408 (4)	−0.0816 (3)	0.045 (2)	
H1	0.57717	0.21391	0.02304	0.0692*	
H4	0.71293	0.00947	0.22552	0.0471*	
H7	1.27375	−0.17112	0.03010	0.0583*	
H8	1.40791	−0.15515	−0.10460	0.0694*	
H9	1.27695	−0.02190	−0.17559	0.0702*	
H10	0.98850	0.09384	−0.11132	0.0537*	

### Atomic displacement parameters (Å^2^)

**Table:**

	*U*^11^	*U*^22^	*U*^33^	*U*^12^	*U*^13^	*U*^23^
Br1	0.0513 (4)	0.0432 (3)	0.0535 (4)	−0.0002 (3)	0.0022 (4)	−0.0087 (3)
Br2	0.0679 (5)	0.0465 (4)	0.0727 (5)	0.0007 (3)	−0.0084 (4)	0.0200 (4)
O1	0.049 (3)	0.034 (2)	0.056 (3)	0.002 (2)	0.002 (2)	0.007 (2)
C1	0.030 (3)	0.036 (3)	0.034 (3)	−0.005 (3)	−0.008 (3)	−0.005 (3)
C2	0.034 (3)	0.030 (3)	0.039 (4)	−0.008 (3)	−0.009 (3)	0.003 (3)
C3	0.033 (3)	0.036 (4)	0.038 (4)	−0.008 (3)	0.000 (3)	−0.006 (3)
C4	0.041 (4)	0.039 (3)	0.038 (4)	−0.008 (3)	−0.006 (3)	0.005 (3)
C5	0.039 (3)	0.037 (3)	0.049 (4)	−0.006 (3)	−0.014 (3)	0.008 (3)
C6	0.033 (3)	0.031 (3)	0.049 (4)	−0.007 (3)	−0.012 (3)	−0.002 (3)
C7	0.047 (4)	0.042 (4)	0.056 (5)	0.004 (3)	−0.017 (4)	−0.012 (4)
C8	0.052 (4)	0.063 (5)	0.059 (6)	0.011 (4)	−0.004 (4)	−0.026 (5)
C9	0.052 (5)	0.081 (6)	0.043 (5)	−0.001 (4)	0.003 (4)	−0.006 (4)
C10	0.042 (4)	0.049 (3)	0.044 (4)	−0.002 (3)	−0.012 (3)	0.001 (3)

### Geometric parameters (Å, °)

**Table:**

Br1—C3	1.898 (6)	C5—C6	1.415 (8)
Br2—C5	1.910 (6)	C6—C7	1.419 (9)
O1—C2	1.377 (7)	C7—C8	1.337 (10)
O1—H1	0.8200	C8—C9	1.374 (10)
C1—C2	1.425 (8)	C9—C10	1.377 (9)
C1—C10	1.409 (7)	C4—H4	0.9300
C1—C6	1.431 (7)	C7—H7	0.9300
C2—C3	1.331 (9)	C8—H8	0.9300
C3—C4	1.391 (8)	C9—H9	0.9300
C4—C5	1.353 (8)	C10—H10	0.9300
			
C2—O1—H1	110.00	C1—C6—C5	116.6 (5)
C2—C1—C6	118.7 (5)	C6—C7—C8	123.4 (6)
C2—C1—C10	122.6 (5)	C7—C8—C9	119.9 (7)
C6—C1—C10	118.7 (5)	C8—C9—C10	120.6 (7)
O1—C2—C1	114.8 (5)	C1—C10—C9	120.8 (5)
O1—C2—C3	124.3 (5)	C3—C4—H4	121.00
C1—C2—C3	120.9 (5)	C5—C4—H4	120.00
Br1—C3—C4	117.9 (5)	C6—C7—H7	118.00
C2—C3—C4	121.7 (6)	C8—C7—H7	118.00
Br1—C3—C2	120.4 (5)	C7—C8—H8	120.00
C3—C4—C5	119.1 (6)	C9—C8—H8	120.00
Br2—C5—C6	119.2 (4)	C8—C9—H9	120.00
C4—C5—C6	122.9 (6)	C10—C9—H9	120.00
Br2—C5—C4	117.8 (5)	C1—C10—H10	120.00
C1—C6—C7	116.6 (5)	C9—C10—H10	120.00
C5—C6—C7	126.9 (6)		
			
C6—C1—C2—O1	179.3 (5)	Br1—C3—C4—C5	−178.8 (4)
C6—C1—C2—C3	−1.5 (8)	C2—C3—C4—C5	−0.9 (9)
C10—C1—C2—O1	1.0 (8)	C3—C4—C5—Br2	176.9 (4)
C10—C1—C2—C3	−179.8 (6)	C3—C4—C5—C6	0.5 (9)
C2—C1—C6—C5	1.0 (8)	Br2—C5—C6—C1	−176.9 (4)
C2—C1—C6—C7	−179.7 (5)	Br2—C5—C6—C7	3.8 (9)
C10—C1—C6—C5	179.4 (5)	C4—C5—C6—C1	−0.5 (9)
C10—C1—C6—C7	−1.3 (8)	C4—C5—C6—C7	−179.8 (6)
C2—C1—C10—C9	179.2 (6)	C1—C6—C7—C8	0.4 (9)
C6—C1—C10—C9	0.9 (9)	C5—C6—C7—C8	179.6 (6)
O1—C2—C3—Br1	−1.5 (8)	C6—C7—C8—C9	1.0 (10)
O1—C2—C3—C4	−179.4 (5)	C7—C8—C9—C10	−1.5 (10)
C1—C2—C3—Br1	179.3 (4)	C8—C9—C10—C1	0.5 (10)
C1—C2—C3—C4	1.4 (9)		

### Hydrogen-bond geometry (Å, °)

**Table:**

*D*—H···*A*	*D*—H	H···*A*	*D*···*A*	*D*—H···*A*
O1—H1···Br1	0.82	2.60	3.107 (5)	122
O1—H1···O1^i^	0.82	2.21	2.893 (6)	141

Symmetry codes: (i) *x*−1/2, −*y*+1/2, −*z*.
